# Integration of transcriptomics and gut microbiomics reveals walnut septum polyphenols alleviate HFD-induced lipid disorders

**DOI:** 10.1038/s41538-026-00801-y

**Published:** 2026-03-26

**Authors:** Yue-Xiu Pan, Lei Peng, Xia Hu, Jin-lian Chen, Min Su, Jing-jing Dai, Jun Sheng, Zi-Shan Hong, Jing Xie, Yang Tian

**Affiliations:** 1 College of Pharmacy, Shan Dong Xian Dai University, Jinan, China; 2 College of Food Science and Technology, Yunnan Agricultural University, Kunming, China; 3 Engineering Research Center of Development and Utilization of Food and Drug Homologous Resources, Ministry of Education, Yunnan Agricultural University, Kunming, China; 4 Yunnan Provincial Key Laboratory of Precision Nutrition and Personalized Food Manufacturing, Yunnan Agricultural University, Kunming, China; 5 School of Tea and Coffee, Puer University, Puer, China; 6 School of Life and Health Technology, Dongguan University of Technology, Dongguan, China

**Keywords:** Obesity, Transcriptomics, Nutrition, Microbial communities

## Abstract

Walnut septum, an underutilized agricultural by-product, exhibits anti-obesity potential. However, the in vivo hypolipidemic mechanisms of walnut septum polyphenols (WSP) remain unexplored. We investigated the effects of WSP on lipid metabolism in high-fat diet (HFD)-fed mice using integrated transcriptomic and gut microbiomic analyses. The results indicated that WSP inhibited lipid accumulation in HFD mice and ameliorated HFD-induced oxidative stress, inflammation, and gut barrier impairment. Further studies revealed that WSP positively regulated FoxO1 expression by suppressing the PI3K/AKT signaling pathway, which in turn inhibited hepatic lipid synthesis in HFD mice. Furthermore, WSP concurrently remodeled gut microbiota *via* selective enrichment of beneficial *Akkermansia* and depletion of inflammation-associated *norank_f__Desulfovibrionaceae*. This microbial shift correlated with enhanced intestinal barrier integrity, reduced endotoxemia, and a predicted upregulation of propanoate metabolism. This study provides the first evidence of the synergistic regulation of the PI3K/AKT/FoxO1 pathway and gut microbiota restructuring by WSP, establishing a scientific foundation for valorizing walnut-processing waste into nutraceuticals against obesity.

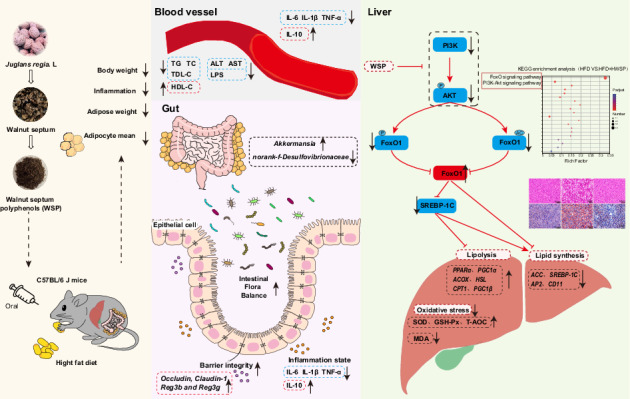

## Introduction

Obesity, characterized by significant overweight and an excessive accumulation of fat, is mainly a result of a chronic mismatch between energy intake and output. It is often associated with dyslipidemia, mild inflammation, oxidative stress, and intestinal dysfunction^1^, and represents a major global health issue and a risk factor for several chronic diseases^1,2^. The clinically approved use of drugs such as orlistat for treating obesity is frequently associated with significant toxic side effects^3^. Therefore, exploring healthier, natural plant-derived pharmaceutical ingredients or dietary supplements for obesity management is of paramount importance.

Lipid synthesis and fatty acid β-oxidation are two key biological processes in lipid metabolism. The important role played by the liver in lipid synthesis and transport has been widely recognized. Studies have shown that HFD induces fatty liver, hepatic steatosis, and leads to hepatic dysfunction in mice^2,4^. Hepatic insulin signaling *via* the PI3K/AKT pathway suppresses gluconeogenesis, lipolysis, and glycogenolysis while enhancing lipogenesis and glycogen synthesis^5^. The PI3K/AKT pathway is located upstream of FoxO1, and PI3K increases the phosphorylation level of AKT, and activated AKT subsequently phosphorylates FoxO1 at multiple sites, negatively regulating FoxO1^6^. Previous research has demonstrated that FoxO1, an important transcription factor affecting adipocyte differentiation, can effectively inhibit adipocyte differentiation^7^. Therefore, suppressing PI3K/AKT signaling and increasing the effective expression of FoxO1represents an effective strategy to inhibit lipid accumulation.

Accumulating scientific data consistently indicate that the composition of gut microbiota is a key factor in obesity and other metabolic dysfunctions. The liver serves as the initial organ exposed to intestinal derivatives, and dysbiosis of the intestinal microbiota is an important cause of liver dysfunction^8^. Obesity often triggers a systemic low-grade inflammatory state and further drives metabolism-related complications. It is noteworthy that intestinal flora disturbances can contribute to the obesity process *via* multifaceted mechanisms. These mechanisms encompass not only the induction of chronic inflammation and disruption of intestinal barrier function, but also the deregulation of host energy metabolism homeostasis and alteration of feeding behavior. Additionally, these mechanisms involve the modulation of circadian rhythm-related gene transcription—all of which are critical factors in the initiation and progression of obesity^2^. Consequently, preserving a healthy gut microbiota is essential for ensuring normal metabolic and energy homeostasis in the body.

Walnut (*Juglans regia* L.) is one of the important nuts in the world, rich in a variety of nutrients and is considered a natural functional food. Walnut septum is the wooden septum in the middle of walnut, which is a common by-product of walnuts. Walnut septum possesses various chemical constituents such as phenolic acids, flavonoids, carbohydrates, and lipid components^9^, and exhibits a variety of bioactivities in vitro and in vivo, including antioxidant activity, antibacterial, anti-inflammatory, antidiabetic, anti-tumor, and anti-aging properties^9,10^. However, Walnut septum is often discarded in food processing, resulting in a waste of resources. Phenolic compounds extracted from walnut septum have demonstrated superior antioxidant and anti-inflammatory activities, and the ability to inhibit α-glucosidase and lipase activities in vitro^3^. Notably, lipase accelerates the hydrolysis of dietary fats, facilitates the digestion of triglycerides, and promotes the absorption of lipids. In addition, as demonstrated in our prior research, it was observed that WSP had a positive effect on hypoglycemia and hypolipidemia via in vitro enzyme inhibition and cellular assays^11^. This suggests that walnut septum polyphenols (WSP) have the potential to inhibit lipid accumulation. However, its in vivo anti-obesity efficacy and mechanisms, particularly regarding gut-liver crosstalk, remain unexplored.

Therefore, this study aimed to systematically investigate the in vivo hypolipidemic effects and underlying mechanisms of WSP in HFD-fed mice, for the first time employing integrated hepatic transcriptomic and gut microbiomic analyses. Our findings offer novel mechanistic insights into how WSP modulates lipid metabolism to facilitate weight reduction, not only advancing our understanding of natural phenolics in lipid homeostasis but also supporting the valorization of walnut septum as a functional ingredient for metabolic health.

## Results

### Effects of WSP on physiological indices in HFD mice

**Fig. 1:**
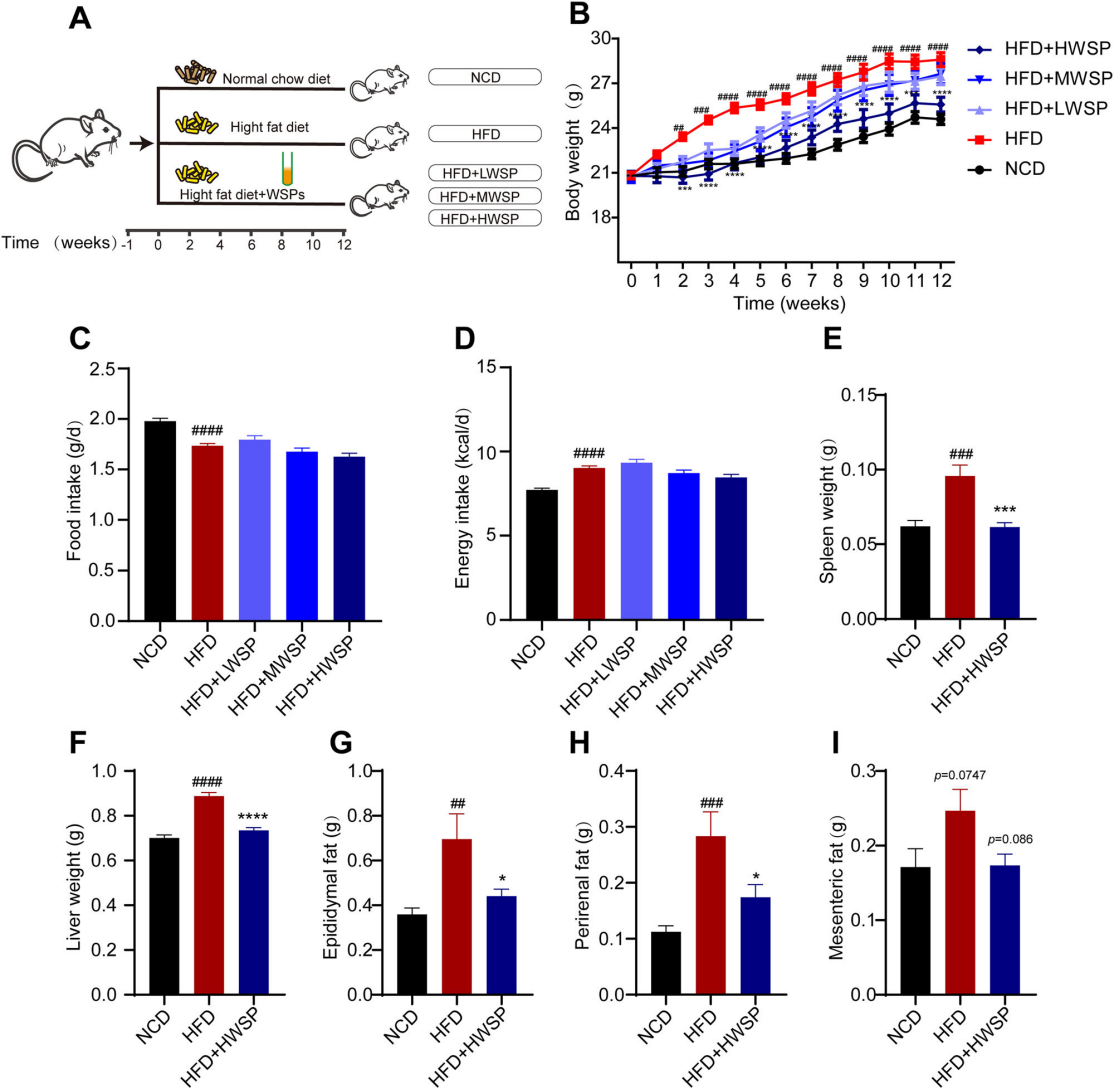
Supplementation with WSP attenuates the adverse effects of HFD on basal indices in mice. **A** Schematic overview of the experimental design. **B** Body weight. **C** Food intake. **D** Energy intake. **E** Spleen weight. **F** Liver weight. **G–I** Epididymal, perirenal, and mesenteric fat weights. (*n* = 12) Data are mean ± SEM. ##*p* < 0.01, ###*p* < 0.001 and ####*p* < 0.0001, *p < 0.05, ***p < 0.001, ****p < 0.0001, # denotes NCD vs. HFD. * denotes HFD vs. HFD + HWSP. Statistical analyses were performed using one-way ANOVA followed by Tukey’s multiple comparison test.

To systematically elucidate the impact of WSP on lipid accumulation in HFD mice, we treated HFD mice with different doses of WSP for 12 weeks (Fig. 1A). Daily observations revealed no adverse signs such as lethargy, piloerection, or mortality in any group. Mice gavaged with HWSP exhibited significant weight loss starting from week 2 (*p* < 0.001), and this reduction persisted until the experiment’s conclusion (Fig. 1B). However, there was no statistical difference between the HFD + LWSP and HFD + MWSP groups compared to the HFD group at the end of the experiment. Notably, energy intake (Fig. 1D) and food consumption (Fig. 1C) remained comparable between HFD-fed mice and WSP-treated groups across all doses. Despite this, high-dose WSP (HWSP, 400 mg/kg) markedly attenuated HFD-induced body weight gain (*p* < 0.001 vs HFD, Fig. 1B), whereas low- and medium-dose interventions exhibited no significant effects. Given the dose-dependent efficacy, HWSP was selected for subsequent mechanistic analyses. HWSP administration significantly reversed HFD-driven increases in liver mass (Fig. 1F), epididymal fat (Fig. 1G), and perirenal fat (Fig. 1H). In addition, splenic weight, a key indicator of immune organ status, was significantly reduced in the HFD + HWSP group compared to HFD controls (*p* < 0.001, Fig. 1E). Furthermore, we found that the HWSP administration did not cause abnormal changes in the organ indices of the heart, lungs, and kidneys, indicating that WSP is non-toxic to mouse organs (*p* > 0.05 vs. NCD, Fig. S1). Collectively, these findings demonstrate that HWSP mitigates obesity-related phenotypes, including adiposity and hepatomegaly, independent of caloric restriction.

### Effects of WSP on serum lipids, inflammatory factors, and transaminase levels in HFD mice

**Fig. 2:**
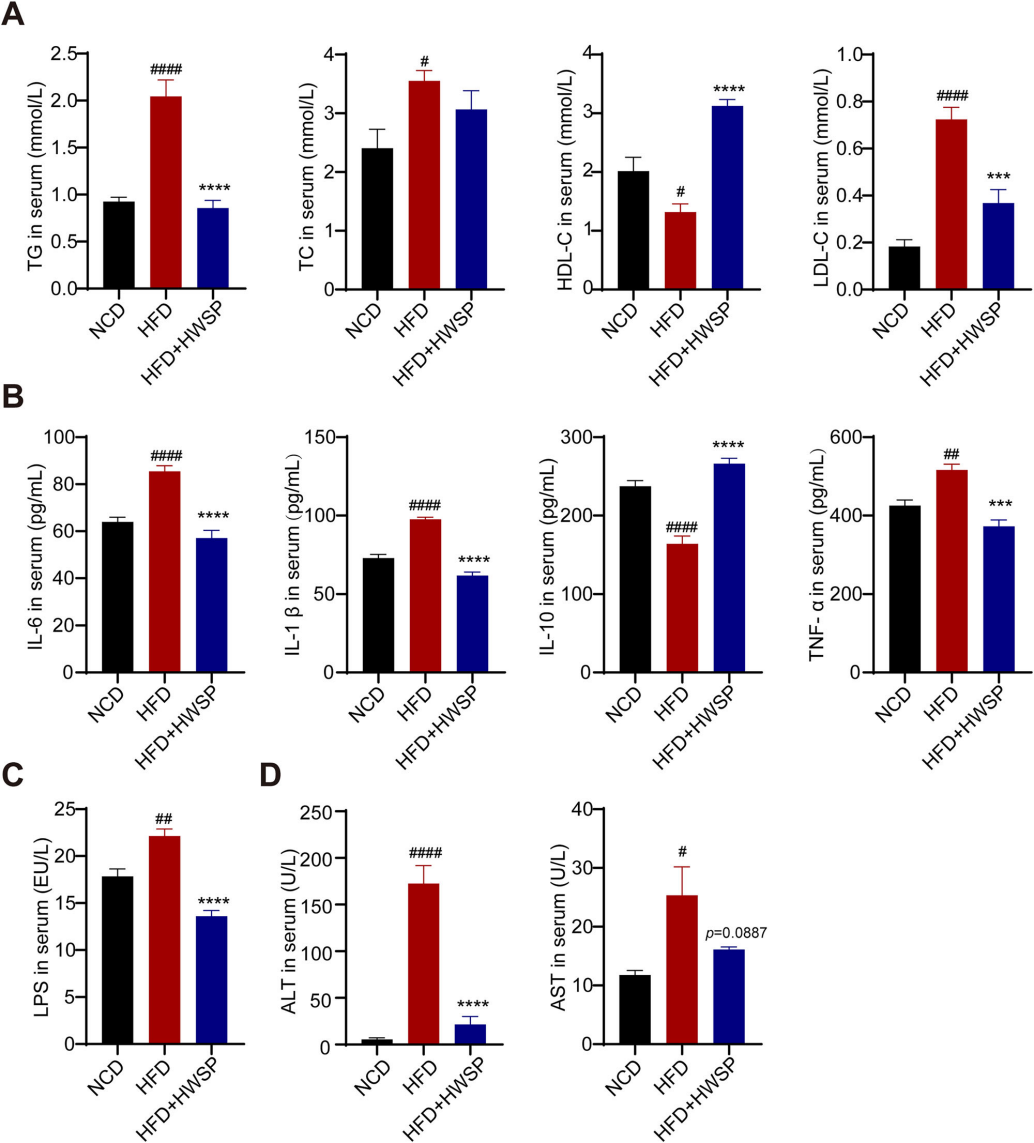
Effect of WSP on serum biochemical indices. **A** Serum lipids including TG, TC, HDL-C, and LDL-C (*n* = 6). **B** Serum levels of IL-6, IL-1β, IL-10 and TNF-α (*n* = 8). **C** Serum LPS (*n* = 8). **D** Serum ALT and AST (*n* = 6). Data are mean ± SEM. #*p* < 0.05, ##*p* < 0.01 and ####*p* < 0.0001, ***p < 0.001, ****p < 0.0001, # denotes NCD vs. HFD. * denotes HFD vs. HFD + HWSP. Statistical analysis was performed with one-way ANOVA followed by Tukey’s multiple comparison test.

HFD mice exhibited significant elevations in serum TG, TC, and LDL-C, alongside a marked reduction in HDL-C relative to the NCD group. Notably, oral administration of HWSP normalized these HFD-induced dyslipidemias (Fig. 2A). Given the well-established link between HFD-induced obesity and systemic inflammation, we assessed serum levels of key inflammatory mediators. Our analysis revealed that HFD significantly upregulated serum levels of pro-inflammatory cytokines (IL-6, IL-1β, and TNF-α), as well as LPS concentrations, while concurrently suppressing anti-inflammatory IL-10 (Fig. 2B, C). Notably, HWSP treatment attenuated this pro-inflammatory shift, as evidenced by a reduction in circulating levels of IL-6, IL-1β, TNF-α, and LPS and an increase in IL-10 concentrations compared with the HFD group (Fig. 2B, C). In addition, we examined the serum levels of ALT and AST, and we found that HWSP significantly reduced the serum levels of AST (*p* < 0.0001) and ALT compared to the HFD group (Fig. 2D). These results demonstrate that WSP effectively reduces serum lipid levels and systemic inflammation, while ameliorating hepatic impairment in HFD-fed mice.

### Effects of WSP on adipocyte size, hepatic lipid metabolism, and oxidative stress in HFD mice

**Fig. 3:**
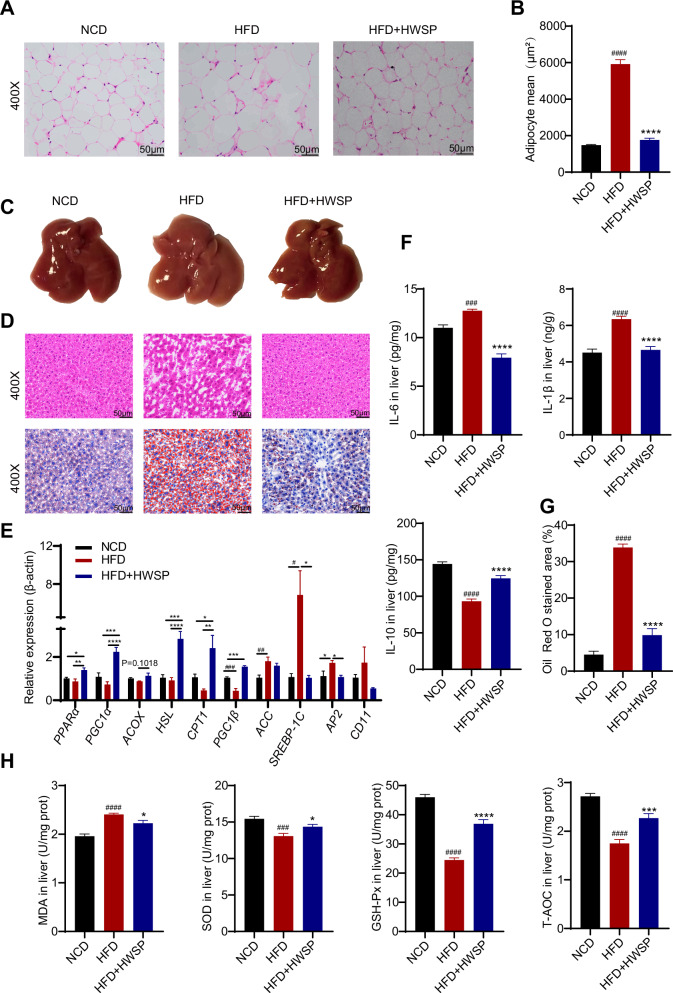
WSP ameliorated HFD-induced abnormal hepatic lipid metabolism and hepatic oxidative stress. **A** Representative micrographs of H&E-stained histological sections of epididymal fat. **B** Average adipocyte area (*n* = 32). **C** Representative photographs of the liver. **D** Representative micrographs of histological sections of liver tissues stained with H&E and Oil Red O. **E** mRNA expression levels of lipid metabolism-related genes in liver tissues (*n* = 6). **F** Liver tissue levels of IL-6, IL-1β, and IL-10. **G** Oil Red O stained area (%, *n* = 12). **H** MDA, SOD, GSH-Px, and T-AOC levels in liver tissues. Data are mean ± SEM. #*p* < 0.05, ##*p* < 0.01, ###*p* < 0.001 and ####*p* < 0.0001, *p < 0.05, **p < 0.01, ***p < 0.001, ****p < 0.0001, # denotes NCD vs. HFD. * denotes HFD vs. HFD + HWSP. statistical analyses were performed using one-way factor ANOVA followed by Tukey’s multiple comparison test.

H&E and Oil Red O staining revealed that WSP treatment alleviated high-fat diet-induced epididymal adipocyte hypertrophy (Fig. 3A, B). Compared with the NCD group, HFD-fed mice exhibited significant hepatic lipid vacuoles, hepatic lobule edema, and steatosis. WSP treatment effectively mitigated these pathological manifestations (Fig. 3C, D, G). Furthermore, ELISA analysis of liver tissues showed that WSP administration attenuated HFD-induced hepatic inflammation (Fig. 3F).

The hepatic mRNA levels of genes involved in lipid metabolism were measured *via* RT-qPCR. WSP treatment reversed the HFD-induced dysregulation of hepatic lipid metabolism by suppressing the expression of lipogenic genes *ACC*, *SREBP-1C* (*p* < 0.05), *AP2* (*p* < 0.05), and *CD11*, while promoting the transcription of genes critical for fatty acid oxidation (*PPARα*, *PGC1α*, *HSL*, *CPT-1*, *PGC1β*) (Fig. 3E). Concurrently, WSP alleviated hepatic oxidative stress in HFD mice, as evidenced by a significant reduction in MDA levels and a marked elevation in GSH-Px, SOD, and T-AOC activities (*p* < 0.001) compared with the HFD group (Fig. 3H). These findings suggest that WSP exerts a protective effect against HFD-induced oxidative stress in the liver.

### WSP attenuated intestinal inflammatory response and improved intestinal barrier function in HFD mice

**Fig. 4:**
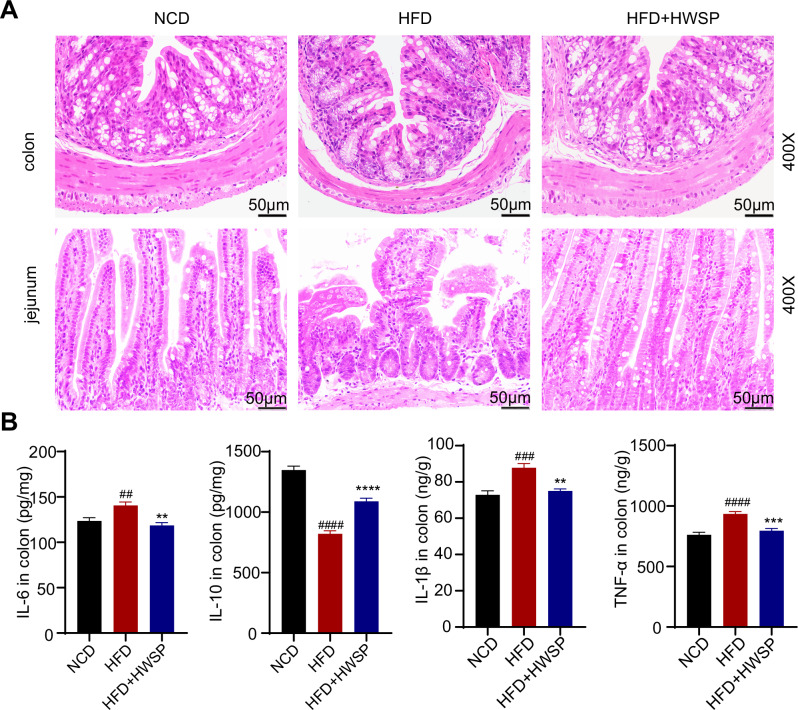
WSP attenuated intestinal inflammatory response and improved intestinal barrier function. **A** Representative micrographs of H&E-stained histological sections of the jejunum and colon. **B** Levels of IL-6, IL-1β, IL-10, and TNF-α in colonic tissues (*n* = 6). Data are mean ± SEM. ##*p* < 0.01 and ####*p* < 0.0001, *p < 0.05, **p < 0.01, ***p < 0.001, ****p < 0.0001, # denotes NCD vs. HFD. * denotes HFD vs. HFD + HWSP or NCD vs HFD + HWSP. Statistical analyses were performed using one-way ANOVA followed by Tukey’s multiple comparison test.

To assess the impact of WSP on intestinal histopathology in HFD mice, we conducted H&E staining of colon and jejunal sections. The results revealed that WSP treatment ameliorated HFD-induced inflammatory cell infiltration, epithelial cell lose, and intestinal villus disruption and shortening (Fig. 4A). Subsequently, we examined the expression levels of inflammation-related cytokines (IL-6, IL-1β, TNF-α, and IL-10) in colon tissues. Consistent with HFD-induced intestinal inflammation, the colonic levels of IL-6, IL-1β, and TNF-α were markedly upregulated, while IL-10 was downregulated in the HFD group (*p* < 0.05). WSP intervention significantly mitigated these cytokine alterations (Fig. 4B). Furthermore, RT-qPCR quantification of colon tissues demonstrated that WSP restored the mRNA expression of *Occludin*, *Claudin-1*, and *Reg3b* in HFD mice (Fig. S2). These data collectively indicate that WSP alleviates intestinal inflammatory responses and enhances the intestinal barrier in HFD-fed mice.

### WSP inhibits lipid accumulation in HFD mice by regulating the PI3K/AKT/FoxO1 signaling

**Fig. 5:**
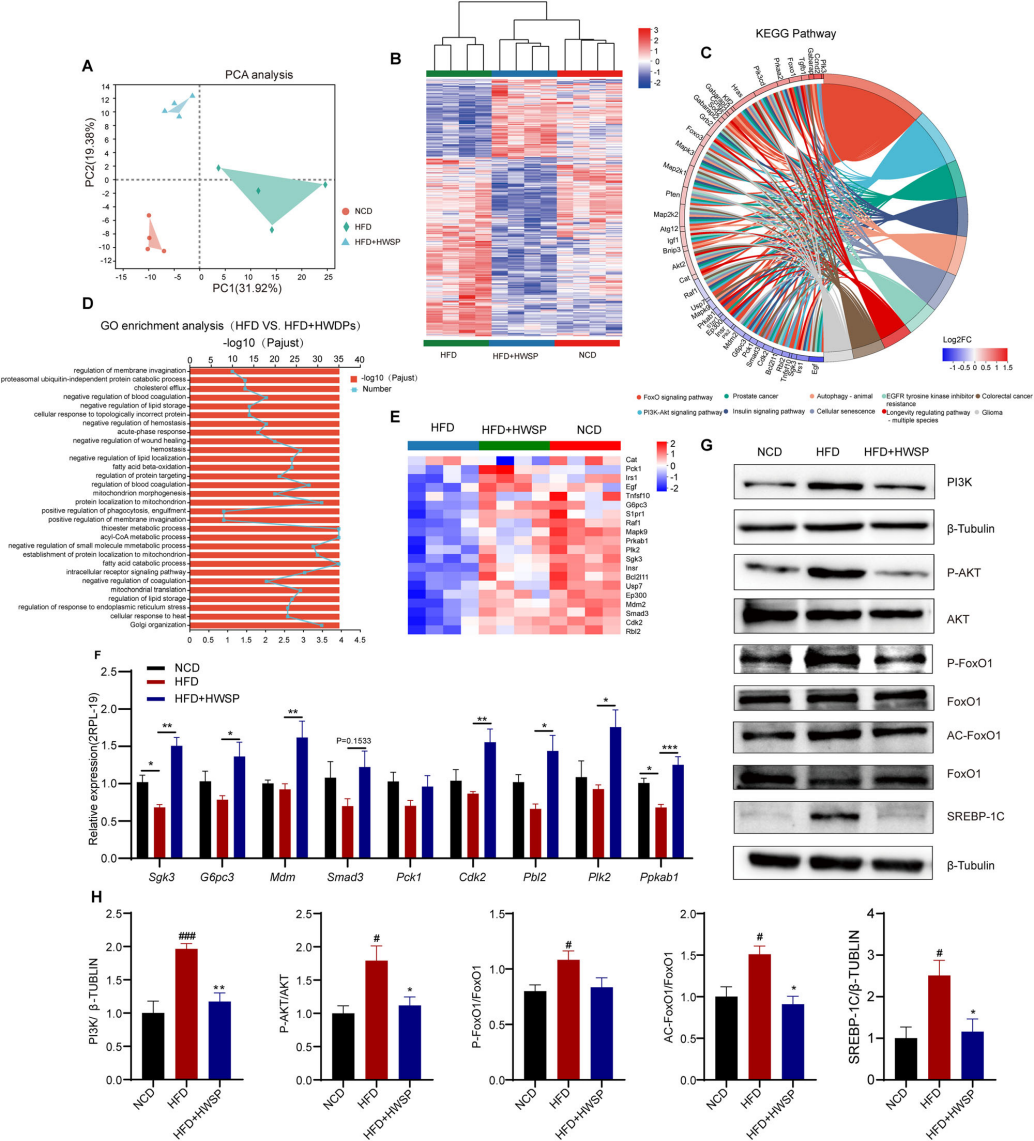
WSP inhibits lipid accumulation in HFD mice by regulating the PI3K/AKT/FoxO1 signaling. **A** Principal component analysis (PCA) among the three groups. **B** Heat map of differentially expressed genes in the three groups. **C** KEGG enrichment analysis of DEGs between the HFD and HFD + HWSP groups. **D** GO enrichment analysis of DEGs between the HFD and HFD + HWSP groups. **E** Heatmap of DEGs in the FoxO1 pathway. **F** Validationg of DEGs in the FoxO1 pathway by qRT-PCR (*n* = 6). **G**–**H** Representative Western blot images and quantification for expression of PI3K (*n* = 5), P-AKT/AKT (*n* = 4), P-FoxO1/FoxO1 (*n* = 6), ACFoxO1/FoxO1 (*n* = 3), SREBP-1C (*n* = 4). Data are mean ± SEM. ##*p* < 0.01 and ####*p* < 0.0001, *p < 0.05, ***p < 0.001, # denotes NCD vs. HFD. * denotes HFD vs. HFD + HWSP or NCD vs HFD + HWSP. Statistical analyses were performed using one-way ANOVA followed by Tukey’s multiple comparison test.

To elucidate the underlying mechanisms by which WSP inhibits lipid accumulation, we performed liver transcriptome assays. Distinct transcriptional patterns were evident across the three groups; notably, the transcriptome profiles of the NCD and HFD + HWSP groups clustered more closely together, indicating that WSP treatment shifted the gene expression pattern toward that of the control mice (Fig. 5A, B). Further analysis has identified 5249 DEGs in the HFD mice versus the NCD mice, comprising 2422 up-regulated and 2827 down-regulated genes. In contrast, the HFD + HWSP group exhibited 6440 DEGs compared to the HFD group, with 3361 up-regulated and 3079 down-regulated genes (Fig. S3). GO analysis demonstrated that these DEGs were significantly associated with biological processes involved in lipid metabolism and storage, such as the negative regulation of lipid storage and lipid localization, fatty acid beta-oxidation, acyl-CoA metabolic process, fatty acid catabolic process, regulation of lipid storage (Fig. 5D). In addition, KEGG enrichment analysis showed that WSP significantly regulated the FoxO signaling pathway and PI3K/AKT signaling in HFD mice (Fig. 5C). Subsequently, genes within the FoxO signaling pathway that showed significant enrichment were selected for expression pattern clustering. The results indicated that the gene expression trends in the WSP-treated group were highly consistent with those in the NCD group (Fig. 5E). Furthermore, RT-qPCR quantification of key genes in these pathways aligned with the transcriptomic data(Fig. 5F).

To determine the regulatory effects of WSP on the PI3K/AKT/FoxO1 signaling, Western blot assays were performed. The protein levels of PI3K, P-AKT, AC-FoxO1, P-FoxO1, and SREBP-1C were significantly upregulated in the HFD group relative to the NCD group (*p* < 0.05), whereas WSP treatment significantly reversed these HFD-induced alterations (Fig. 5G). Our findings demonstrated that WSP suppresses lipid accumulation in HFD mice *via* modulation of the PI3K/AKT/FoxO1 signaling.

### WSP ameliorates HFD-induced gut microbiota dysbiosis and functional disorders

**Fig. 6:**
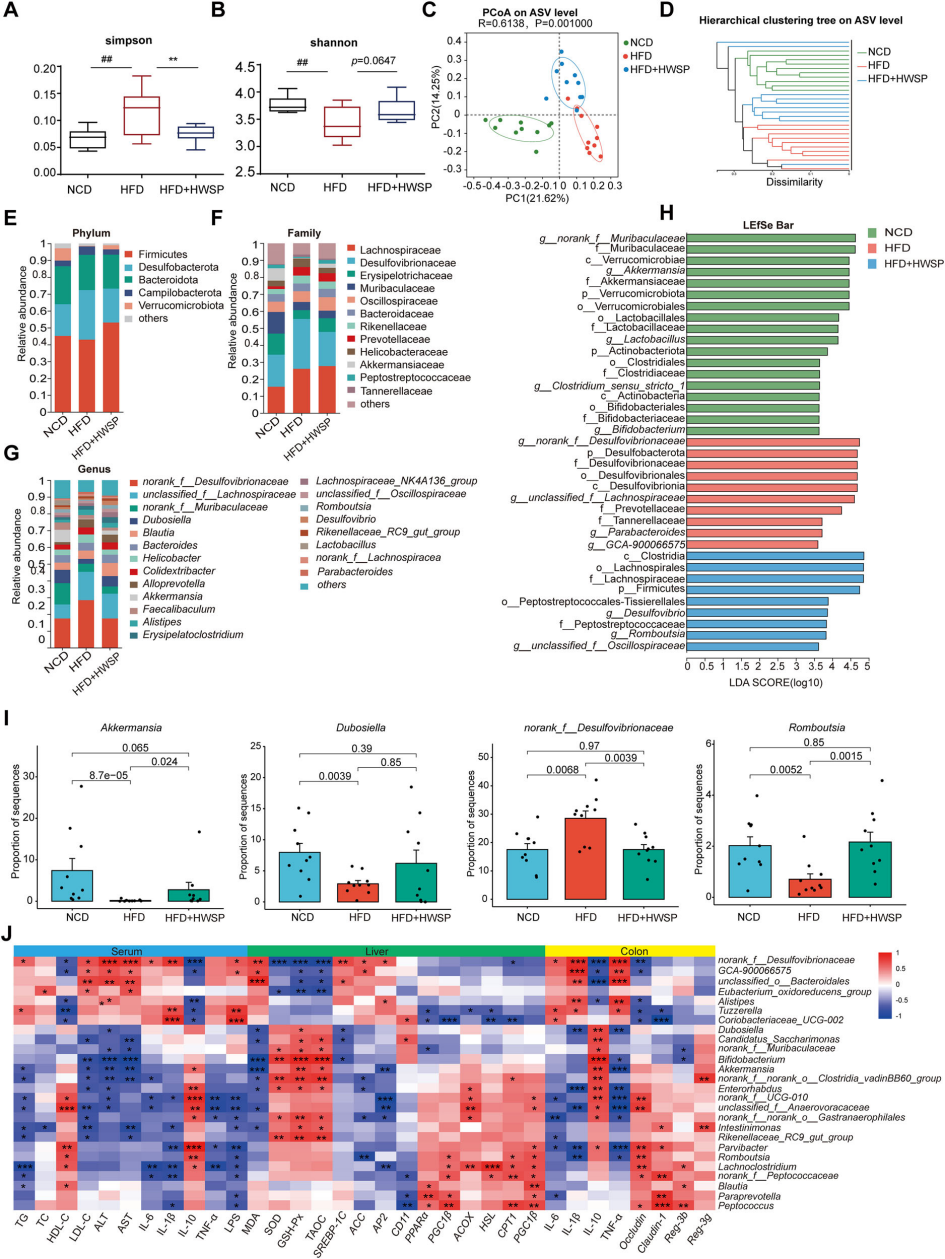
WSP ameliorated HFD-induced intestinal flora disorders. **A**, **B** Alpha diversity analysis (Simpson and Shannon indices) of the gut microbiota. **C**, **D** Principal coordinate analysis (PCoA) plots based on the Bray-Curtis distance matrix and a hierarchical clustering tree evaluating the microbial community composition at the ASV level. Structural composition and comparison of relative abundance at the phylum (**E**), family (**F**) and genus (**G**) levels. **H** Analysis based on LEfSe (LDA discriminant bar graph). **I** Comparison of representative microbial genera relative abundance among the NCD, HFD, and HFD + HWSP groups. **J** Spearman’s correlation analysis between the gut microbiota and obesity-related variables. (*n* = 10) Data are mean ± SEM. #*p* < 0.05, ##*p* < 0.01, ###*p* < 0.001 and ####*p* < 0.0001, *p < 0.05, **p < 0.01, ***p < 0.001, ****p < 0.0001, # denotes NCD vs. HFD. * denotes HFD vs. HFD + HWSP or NCD vs HFD + HWSP. Statistical analyses were performed using one-way ANOVA followed by Tukey’s multiple comparison test.

The modulatory impact of WSP on gut microbiome structure and community composition was examined. We found that WSP administration was associated with a significant decrease in the Simpson index (*p* < 0.01) and a concomitant increase in the Shannon index in HFD-fed mice, indicating enhanced microbial diversity (Fig. 6A, B). Furthermore, PCoA and hierarchical clustering analysis revealed that samples from the three groups were distinctly separated along the principal axes, demonstrating significant differences in microbial community composition (Fig. 6C, D). At the phylum level, the cecal microbiota was predominantly composed of Firmicutes, Desulfobacterota, and Bacteroidota. HFD-induced reductions in the abundance of Firmicutes and Verrucomicrobiota, alongside increases in Desulfobacterota and Campylobacteria, were significantly reversed by WSP treatment (Fig. 6E). At the family level, HFD-fed mice exhibited an altered microbial profile characterized by an enrichment of Desulfovibrionaceae, Helicobacteraceae, and Tannerellaceae, and a depletion of Erysipelotrichaceae, Akkermansiaceae, Peptostreptococcaceae, and Eggerthellaceae;these changes were effectively mitigated by WSP (Figs. 6F and S4). At the genus level, the proportions of norank_f_*Desulfovibrionaceae*, unclassified_f_*Lachnospiraceae, Helicobacter*, *Parabacteroides*, and GCA-900066575 were increased, and the proportions of *Dubosiella*, *Akkermansia*, unclassified_f_*Oscillospiraceae, Romboutsia, and Enterorhabdus* were decreased in the HFD group, which was reversed following WSP administration (Figs. 6G, I and S5).

LEfSe analysis identified 43 distinct microbial taxa that differed significantly across the three mouse groups, including 5 phyla, 6 classes, 9 orders, 11 families and 12 genera. A total of 37 linear ASVs were obtained for the three groups (Fig. 6H). Furthermore, we found that *Desulfovibrionaceae* (from phylum to genus), Parabacteroides (from Tannerellaceae family to *Parabacteroides*) and Prevotellaceae family were the dominant organisms in the HFD group, and the enrichment of these organisms may serve as a pivotal factor contributing to the dysbiosis of gut microbiota in HFD mice. *Romboutsia* (from the order Peptostreptococcales-Tissierellales to the genus *Romboutsia*) and Lachnospiraceae (from the order Lachnospiracea to the Lachnospiraceae) were significantly enriched in the WSP group. These findings indicate that WSP can ameliorate HFD-induced gut microbial imbalance in mice.

Spearman’s correlation analysis was conducted to examine associations between gut microbiota composition and obesity-related metrics (Fig. 6J). The results showed that *norank__f__Muribaculaceae, Rikenellaceae_RC9_gut_group, Bifidobacterium, Enterorhabdus, norank__f__norank__o__Gastranaerophilales* were significantly positively correlated with antioxidant factors (SOD, GSH-Px, T-AOC), while *norank__f__Desulfovibrionaceaeand Eubacterium_oxidoreducens_group* showed a significant negative correlation with these antioxidant factors. *Norank__f__Desulfovibrionaceae, GCA-900066575, and unclassified__o_Bacteroidales* showed a significant positive correlation with serum levels of LDL-C, ALT, AST, IL-1β and TNF-α in colon tissues, whereas they showed a significant negative correlation with IL-10 levels. Similarly, *norank__f__UCG-010* and *Lachnoclostridium* showed a significant negative correlation with serum cytokines (IL-6, IL-1β, TNF-α) and a significant positive correlation with IL-10 levels. In addition, we found that *Peptococcus, Paraprevotella, Blautia, norank__f__Peptococcaceae, Lachnoclostridium, Romboutsia, and Parvibacter* were positively correlated with the expression levels of lipid-degradation-related genes (*PPARα, PGC1α, ACOX, HSL, CPT1, PGC1β*) and barrier-associated proteins (*Occludin, Claudin-1, Reg-3b, Reg-3g*), whereas they were negatively correlated with *SREBP-1C, AP2, and CD11*. These results indicated that WSP improved the HFD-induced intestinal microbiota disorders in mice, which indicate that WSP had a modulating effect on inflammatory response, intestinal barrier, lipid metabolism, and blood lipid levels in HFD mice.

**Fig. 7:**
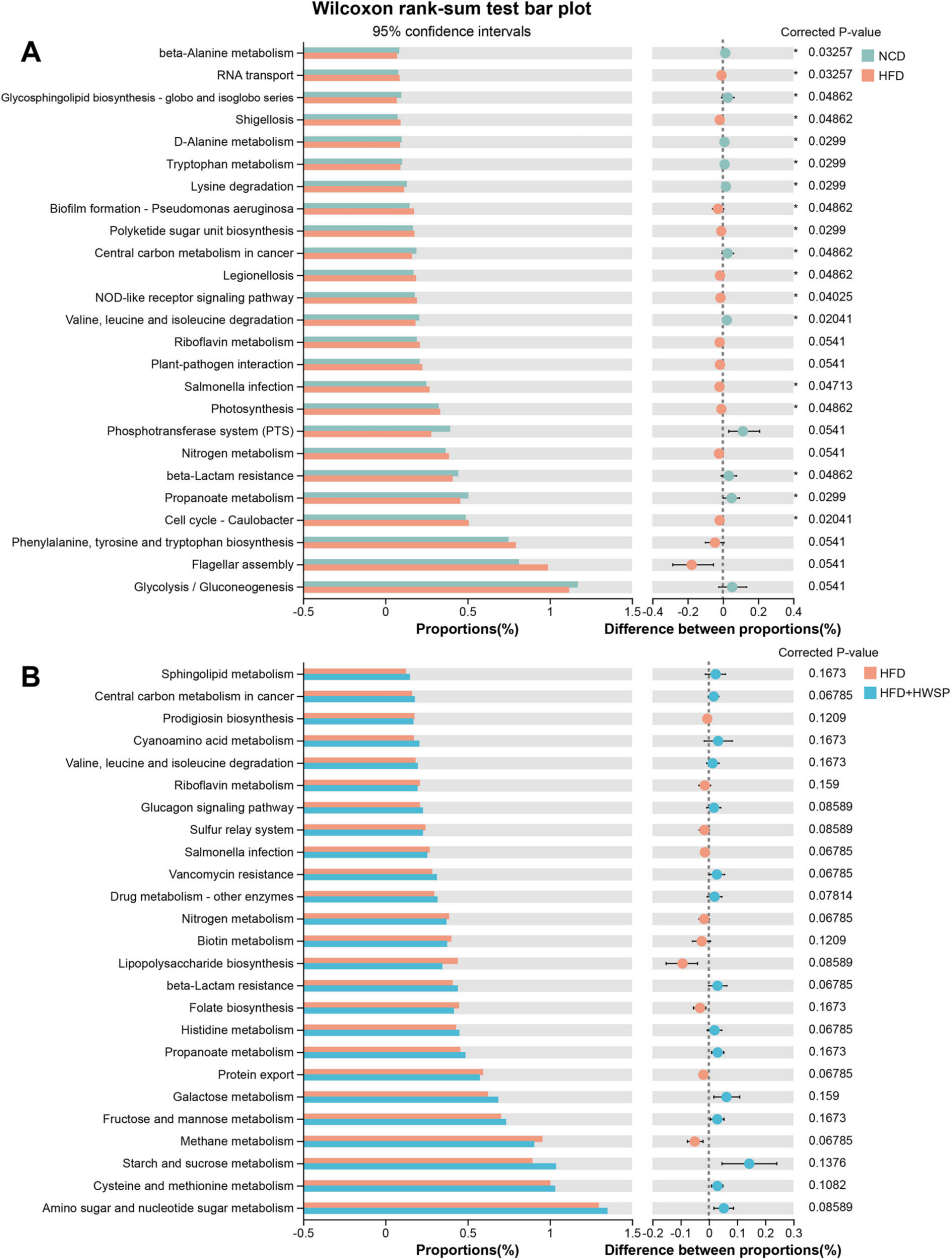
PICRUSt-predicted functions of the gut microbiota in mice. **A** NCD group vs. HFD group. **B** HFD group vs. HFD + HWSP group. Statistical analysis was performed using Mann-Whitney U test. *n* = 10.

To predict changes in microbial metabolic functions, we performed PICRUSt2 analysis. At level 2, there are 36 metabolic pathways in total. Among them, “metabolism” represents the most crucial metabolic pathway within the gut microbiota, with carbohydrate metabolism being the predominant type, followed by amino acid metabolism (Supplementary Fig. S6). Further analysis was carried out on these pathways at the third level. As shown in Fig. 7A, compared with the NCD group, pathways such as Flagellar assembly, Nitrogen metabolism, and Salmonella infection were significantly upregulated in the HFD group, while Glycolysis/Gluconeogenesis and Propanoate metabolism were notably downregulated. After treatment with WSP, Amino sugar and nucleotide sugar metabolism, along with Propanoate metabolism, increased, whereas Lipopolysaccharide biosynthesis was significantly downregulated (Fig. 7B). Therefore, the increase in Propanoate metabolism levels and the decrease in Lipopolysaccharide biosynthesis levels following WSP treatment may contribute to the amelioration of metabolic disturbances in HFD-fed mice.

## Discussion

Obesity is a common metabolic disorder that predisposes individuals to various chronic diseases, with its incidence increasing annually. Its pathogenesis involves complex factors, including energy metabolism imbalance, chronic inflammation, oxidative stress, impaired intestinal barrier function, and gut microbiota dysbiosis. Natural polyphenols have shown promise in mitigating these issues. However, although walnut septum is a unique and underutilized agricultural by-product with great potential, the specific role and comprehensive mechanism by which WSP exert lipid-lowering effects remain largely unexplored. Our study demonstrates that WSP inhibited lipid accumulation, ameliorated inflammatory responses and oxidative stress, and improved the intestinal barrier in HFD-fed mice. Crucially, integrating transcriptomic and gut microbiome analyses, we reveal for the first time that WSP orchestrates a dual regulatory mechanism: it directly modulates hepatic lipid metabolism *via* the PI3K/AKT/FoxO1 axis and concurrently reshapes the gut microbiota composition, particularly enriching beneficial *Akkermansia* and suppressing detrimental *norank_f_Desulfovibrionaceae*. This integrated gut-liver axis approach provides a more comprehensive understanding of WSP’s anti-obesity mechanism than isolated pathway studies.

Significant progress has been made in the study of polyphenols in lipid-lowering. For example, polyphenols from fu brick tea effectively attenuated HFD-induced obesity in rats *via* modulation of gut microbial community dynamics, intestinal oxidative stress, and epithelial barrier integrity^1^. Similarly, dietary polyphenolic compounds, including pomegranate peel polyphenols^12^, and green pea hull polyphenol^13^, have demonstrated pronounced hypolipidemic activities. Critically, the efficacy of polyphenols is inherently dose-dependent. To determine the safety of this dosage, we calculated the organ indices of the heart, lungs, and kidneys in mice. We found that the high dose (400 mg/kg) of WSP did not cause abnormal changes in these indices, indicating that WSP is non-toxic to mouse organs. Our dose-response study identified 400 mg/kg BW/d as the effective dose for WSP in significantly ameliorating HFD-induced lipid disorders, which is consistent with the dose-dependency observed in other phenolic compounds^14^. When translated to human equivalents, the dose is approximately 32 mg per kg of body weight per day, or 1920 mg per day for a 60 kg adult. This dosage falls within the range of high-dose polyphenol interventions that have been demonstrated to be safe and effective in clinical trials targeting metabolic syndrome^15^. While achieving such doses through conventional dietary intake alone may be challenging, it does not diminish the therapeutic potential of WSP. Instead, future research should explore enhancing its translational applicability by optimizing bioavailability or through synergistic formulations with other bioactive compounds, such as dietary fiber or prebiotics.

Obesity is commonly linked to a pro-inflammatory state and exacerbated oxidative stress^16^. Research consistently demonstrates the capacity of polyphenols to suppress inflammation and counteract oxidative stress. For example, polyphenols from green pea hulls^13^, *Poria cocos* tea^1^, cherries^17^, and various other plants have been shown to ameliorate HFD-induced low-grade inflammation and oxidative stress. In our study, we found that WSP significantly restored the balance of inflammatory cytokine and mitigated oxidative stress responses. These results are consistent with prior studies on polyphenolic compounds and further support the potential of polyphenols in obesity intervention. The liver and adipose tissue serve as primary metabolic hubs. Severe obesity disrupts adipocyte function, promoting lipid spillover from adipocytes into the bloodstream and subsequent ectopic lipid accumulation in the liver, resulting in impaired liver function and hepatic steatosis^18^. Consequently, we measured serum ALT and AST levels and found that WSP reversed the HFD-induced elevation of these transaminases. Additionally, our pathological analysis revealed that WSP effectively reduced hepatic inflammation and alleviated vacuolation, steatosis, and lipid droplet accumulation. This indicates that WSP possesses a preventive effect against hepatic dysfunction-associated fatty liver disease.

Previous studies indicate that the downregulation of hepatic lipolytic enzymes (*HSL, ACOX, CPT-1*) and transcriptional coactivators (*PPARα, PGC1α, PGC1β*) is a key feature of HFD-induced obesity^19^. In our study, WSP upregulated the expression levels of these genes to varying degrees, suggesting that WSP stimulates mitochondrial and peroxisomal fatty acid β-oxidation, increases free fatty acid catabolism, and prevents de novo lipogenesis and lipotoxicity^20^.

Disruption of intestinal barrier function and increased intestinal inflammation are critical mechanisms underlying obesity and associated metabolic disorders. Obese individuals often exhibit increased intestinal permeability, allowingendotoxins (e.g., LPS) to enter the circulation and trigger systemic inflammation^21^. Furthermore, HFD-induced obesity can significantly disrupts intestinal barrier structure: on the one hand, it downregulates the transcription of pivotal tight junction (TJ) proteins (*Occludin, Claudin-1*), thereby compromising epithelial integrity and eliciting a surge in pro-inflammatory mediators^22,23^. On the other hand, it inhibits antibacterial peptides such as *Reg3g* and *Reg3g,* which are essential components of the intestinal chemical barrier^24,25^, thus weakening the homeostatic regulation of the bacterial flora^26^, and forming a vicious circle of “barrier damage-inflammation amplification”. Polyphenolic compounds have shown significant effects in protecting the intestinal barrier and reducing intestinal inflammation. For example, polyphenols from fu brick tea were shown to reinforce colonic epithelial integrity by upregulating the transcriptional and translational levels of TJ proteins and suppressing colonic pro-inflammatory cytokine profiles^1^. In contrast, black chokeberry polyphenols alleviate TJ disruption in colon tissues *via* differential regulation of TJ proteins at both mRNA and protein levels, while mitigating obesity-associated colonic inflammation by suppressing the TLR4/NF-κB signaling cascade^23^. Therefore, further experiments have shown that WSP can maintain intestinal barrier homeostasis and attenuate abnormal inflammatory cascades. Additionally, H&E staining of the colon and jejunum revealed that WSP improved HFD-induced inflammatory infiltration and villus shortening in the small intestine. This result is consistent with other studies on polyphenols and further confirms the important role of polyphenols in protecting gut health and alleviating obesity-related metabolic disorders. In addition, the results of the present study further suggest that the preventive effect of WSP against lipid accumulation may be related to the reduction of serum LPS.

In our study, liver transcriptomics via RNA-Seq was employed to identify DEGs among the three groups. Enrichment analysis of DEGs between the HFD and HFD + HWSP groups revealed that the biological processes were predominantly related to lipid metabolism, with the FoxO signaling pathway being the most significantly enriched.. The FoxO transcription factor subfamily, comprising FoxO1, FoxO3, FoxO4, and FoxO6 isoforms, consists of critical transcription factors that regulate metabolic homeostasis^27^. Among them, FoxO1 exerts a pivotal influence on metabolic disorders like obesity by regulating adipocyte differentiation, oxidative stress defense, and the expression of lipid metabolism-related gene ^27,28^. Specifically, FoxO1 can inhibit the transcriptional activity of SREBP-1c promoter by directly binding to it, thereby regulating hepatic glycolipid metabolism^29^. In addition, FoxO1 transcriptional activity is dynamically modulated by phosphorylation and acetylation: phosphorylation promotes its nuclear export and represses transcription, whereas acetylation impairs its DNA-binding ability; these two modifications synergistically regulate FoxO1 function^30^. Notably, the dephosphorylation of FoxO1 negatively regulates adipocyte differentiation, suggesting that its phosphorylation status is critical for lipid metabolic homeostasis^31^. The upstream PI3K/AKT signaling pathway inhibits FoxO1 activity through AKT-mediated phosphorylation, thereby reducing SREBP-1C expression to decrease lipid synthesis. Similarly, Ziziphus jujuba Mill. leaf extract exerts anti-adipogenic effects by suppressing the PI3K/AKT signaling^32^, and Orientin from Commelina communis reduces intracellular lipogenesis by inhibiting the PI3K/AKT signaling at the early stage of adipogenesis^33^, followed by a decrease in FoxO1 phosphorylation. Fucosterol inhibits adipogenesis in 3T3-L1 preadipocytes by down-regulating the insulin-triggered PI3K/Akt pathway and simultaneously decreasing the expression of phosphorylated FoxO1, leading to FoxO1 activation. In addition, Yi Jiao et al. found that adenovirus type 36 could regulate glycolipid metabolism *via* the PI3K/AKT/FoxO1/PPARγ signaling cascade, and that the PI3K-specific inhibitor, Wortmannin, diminished the levels of P-AKT and P-FoxO1, consequently suppressing adipocyte differentiation^7^. In our study, we observed that high-fat diet significantly increased the expression of PI3K, P-AKT, AC- FoxO1, P- FoxO1, and SREBP-1C proteins in the livers of mice, whereas the treatment with WSP significantly reversed the expression of PI3K, P-AKT, AC-FoxO1, and SREBP-1C but did not achieve significant inhibition of P-FoxO1 expression. This suggests that WSP may inhibit lipid accumulation in HFD-fed mice by suppressing the PI3K/AKT pathway, thereby activating the FoxO signaling pathway and enhancing FoxO1 transcriptional activity, which in turn inhibits SREBP-1C expression and reduces lipid synthesis. This mechanism is similar to that of other natural products, further revealing the molecular mechanism by which WSP exert lipid-lowering effects *via* modulating the PI3K/AKT/FoxO1 signaling.

The progression of obesity is frequently accompanied by a disruption of gut microbiota homeostasis and a decline in microbial diversity. In recent times, there has been mounting evidence indicating that an imbalance in the homeostasis, as an important factor contributing to diet-induced obesity, is implicated in related metabolic dysfunctions, and restoring this balance can help ameliorate intestinal inflammation, enhance intestinal barrier integrity, and alleviate intestinal oxidative stress^1^. There has been a gradual increase in studies on the hypolipidemic effects of polyphenolic compounds through modulation of intestinal flora. For example, polyphenols from fu brick tea exhibit anti-obesity activities by improving the abundance of gut microbiota and certain core microorganisms, including *Akkermansia muciniphila*, *Bacteroides*^1^. Tea polyphenols remodeled the gut flora composition and improved gut health by raising the population of probiotic bacteria *Bacteroides*, *Faecalibacterium*, *Paracoccidioides*, and *Akkermansia*^4^.Consistent with these prior findings, our study demonstrates that WSP significantly modulates the structure and function of the gut microbiota. At the community level, the HFD significantly reduced gut microbiota α-diversity (increased Simpson index, decreased Shannon index), indicating diminished bacterial richness and suggesting dysbiosis^1^. Notably, WSP treatment restored α-diversity, highlighting its capacity to profoundly restructure the microbial community. At the taxonomic level, WSP improved lipid homeostasis by promoting the enrichment of beneficial bacteria *Akkermansia*, *Dubosiella*, *Romboutsia* and depleting the detrimental taxon *norank_f__Desulfovibrionaceae*. Specifically, *Akkermansia*, a well-established mucin degrader and promoter of gut barrier function^1^, was significantly enriched in the WSP group. Its abundance showed positive correlations with intestinal barrier-associated proteins and negative correlations with pro-inflammatory cytokines. This suggests *Akkermansia* may restore intestinal epithelial integrity by upregulating tight junction protein expression. Such enhanced barrier function would reduce the translocation of pro-inflammatory bacterial products, particularly LPS, into systemic circulation. This interpretation is supported by our data showing reduced serum LPS levels and attenuated systemic inflammation in WSP-treated mice. Metabolic endotoxemia, a trigger for hepatic insulin resistance and inflammation^34^, can directly or indirectly disrupt lipid metabolism pathways like PI3K/AKT/FoxO1. Therefore, by enriching *Akkermansia*, WSP likely alleviates HFD-induced endotoxemia, thereby mitigating a key inflammatory insult that disrupts hepatic lipid homeostasis. Furthermore, PICRUSt2 functional prediction analysis revealed that WSP-treated mice exhibited a significantly enhanced potential for short-chain fatty acid (SCFA) biosynthesis, particularly propanoate metabolism, compared to the HFD group. This predicted functional shift positively correlated with the WSP-induced enrichment of *Akkermansia*^35^ and other SCFA-producing genera *Dubosiella*^36^, *Romboutsia*^37^. SCFAs are potent signaling molecules influencing host metabolism via activation of G protein-coupled receptors (GPR41, GPR43) and inhibition of histone deacetylases (HDAC)^38^. Critically, butyrate and propionate have been shown to enhance fatty acid oxidation and suppress hepatic lipogenesis by activating PPARα^39^. Although direct measurement of SCFA levels is required for validation, this predicted increase in SCFA production capacity offers a plausible functional link between the WSP-remodeled microbiota and the observed improvements in hepatic lipid metabolism.

Conversely, the significant depletion of inflammation-associated *norank_f__Desulfovibrionaceae* by WSP represents a crucial factor underlying the observed metabolic improvements. *Desulfovibrionaceae* are Gram-negative sulfate-reducing bacteria capable of LPS production^40^. Their overgrowth is closely linked to gut barrier dysfunction, increased intestinal permeability, elevated systemic LPS levels, and the promotion of chronic low-grade inflammation^41^. Correlation analysis confirmed that the abundance of *norank_f__Desulfovibrionaceae* showed significant positive correlations with serum LPS, pro-inflammatory cytokines (IL-6, IL-1β, TNF-α), hepatic oxidative stress (MDA), dyslipidemia markers (TG, LDL-C), and liver injury indicators (ALT, AST). Additionally, functional prediction indicated a downregulation of Lipopolysaccharide biosynthesis pathways in the WSP group, directly corresponding to the significant depletion of *Desulfovibrionaceae* and reduced serum LPS. Thus, the selective inhibition of this detrimental taxon by WSP attenuates a major source of pro-inflammatory and metabolic disruption signals, alleviating inflammation-induced lipid metabolic disorders.

The WSP-mediated remodeling of the gut microbiota fosters a more favorable metabolic milieu by ameliorating intestinal barrier dysfunction, metabolic endotoxemia, and systemic/hepatic inflammation. Substantial evidence confirms that reduced inflammation and endotoxemia improve hepatic insulin sensitivity^42,43^. Improved insulin signaling would be expected to modulate the PI3K/AKT pathway. Our study demonstrated that WSP suppressed HFD-induced hyperactivation of the PI3K/AKT signaling pathway. Although the direct causal links between specific microbial shifts and this pathway require further investigation, the mitigation of microbiota-induced inflammation and endotoxemia by WSP likely functions by restoring hepatic insulin signaling sensitivity, thereby influencing the PI3K/AKT/FoxO1 signaling cascade and ultimately inhibiting lipogenesis.

In summary, this study elucidates that WSP combats HFD-induced obesity and lipid metabolic disorders through a dual-target regulatory strategy. The results demonstrated that WSP was effective in preventing HFD-induced weight gain without altering energy intake in mice. WSP ameliorated HFD-induced abnormalities, including dyslipidemia, hepatic function impairment, dysregulated hepatic energy metabolism, hepatic oxidative stress, hepatic steatosis, epididymal adipocyte hypertrophy, inflammatory responses, intestinal barrier damage, and intestinal microecological dysregulation. Mechanistically, in the liver, WSP suppresseed the PI3K/AKT pathway, thereby enhancing FoxO1 functional activity and inhibiting SREBP-1c-mediated lipogenesis. Concurrently, in the gut, WSP reshapes the gut microbiota by enriching beneficial bacteria (*Akkermansia*, *Dubosiella*, *Romboutsia*) and depleting the harmful bacterium *norank_f__Desulfovibrionaceae*. This microbial restructuring likely improves the inflammatory environment that contributes to hepatic lipid accumulation by enhancing intestinal barrier function, reducing endotoxemia and inflammation, and potentially modulating microbial SCFA metabolism. These gut-centric effects act synergistically with the direct signaling regulation within the liver. In summary, the present study demonstrated that WSP holds great promise as a potential candidate for obesity prevention. These findings not only offer a theoretical foundation to support the application of WSP but also open new perspectives for exploring novel therapeutic strategies for lipid lowering. Future studies should quantify SCFA levels to futther elucidate WSP’s regulatory mechanisms microbial metabolites and employ additional experimental designs to precisely delineate the causal mechanisms underlying WSP-mediated anti-obesity effects.

## Methods

### Walnut septum polyphenols (WSP) preparation

Walnut septum was taken from Yangbi walnut in Dali, Yunnan Province. The walnut septum was crushed using a milling machine and sieved through 60-mesh sieve, according to the parameters of WSP extraction process determined in the previous experiments^11^, ethanol ultrasonication-assisted extraction was carried out to extract polyphenols at the ratio of material to liquid (g: mL) 1:80, pH 5.5, ultrasonic temperature 45 °C, and ultrasonic time of 60 min, and then centrifugation was carried out to collect the supernatant. The crude extract was purified by D101 macroporous resin, eluted with 30% ethanol, and the purity of walnut septum polyphenols reached 87.35%, and the WSP were obtained. The dry powder of WSP was obtained by vacuum freeze-drying, and was stored at –20 °C for further use.

### Animals

A total of 60 male C57BL/6J mice (6 weeks old) were obtained from Hunan Slake Laboratory Animal Co., Ltd. (License No.: SCXK (Xiang) 2019-0004) and maintained in a SPF facility under controlled conditions: temperature 22–26 °C, humidity 55 ± 5%, and a 12-h light/dark cycle. Animals had ad libitum access to food and water throughout the study. All experimental procedures were approved by the IACUC of Yunnan Agricultural University (Approval No. YNAU-202303070).

Following acclimatization period, mice were randomly assigned to five groups (*n* = 12). Normal control diet (NCD): Standard chow (3.9 kcal/g, 10% fat energy; TP23522) + sterile water gavage. High-fat diet (HFD): HFD (5.2 kcal/g, 60% fat energy; TP23520) + sterile water gavage (Supplementary Table S1). HFD + LWSP: HFD + 100 mg/kg BW WSP. HFD + MWSP: HFD + 200 mg/kg BW WSP. HFD + HWSP: HFD + 400 mg/kg BW WSP. The WSP dosages were derived from previous studies and our preliminary experiments^10,44,45^.

### Sample collection

Upon completion of the 12-week intervention, mice were fasted overnight, anesthetized with isoflurane, and unconsciousness was confirmed by the absence of pedal withdrawal reflex. Then, animals were euthanized with CO_2_, and death was confirmed through the absence of corneal reflex and cessation of heartbeat. Thereafter, the abdominal cavity was opened, and blood was collected by puncturing the abdominal aorta with a sterile syringe. Serum was isolated by centrifugation (3500 rpm, 10 min, 4 °C). Major organs (liver, spleen) and adipose tissues (epididymal, perirenal, mesenteric) were dissected and weighed, along with intestinal segments (colon, jejunum, ileum). Liver specimens were processed as follows: one lobe was embedded in OCT compound and snap-frozen in liquid nitrogen (LN₂), while the remaining tissue was directly immersed in LN₂. All hepatic samples were transferred to a −80 °C freezer for long-term preservation. Other tissues were divided into two batches: one batch was flash-frozen in LN₂ (−80 °C storage), and the other was fixed in 4% paraformaldehyde (PFA) for histological examination. Cecal contents were collected aseptically into PBS-containing tubes, rapidly frozen in LN₂, and archived at −80 °C.

### Biochemical assessment

The levels of TC, TG, HDL-C, LDL-C, AST, and ALT were determined by commercially available standard kits (Nanjing Jiancheng Bioengineering Institute, Nanjing, China). An ELISA kit (Jiangsu Meimian Industrial Co., Ltd, Jiangsu, China) was used to detect the levels of IL-1β, IL-6, IL-10, TNF-α, and LPS in the serum and colon, liver tissues of mice. Commercially available standard kits were used to determine the levels of MDA, SOD, GSH-Px, and total T-AOC in liver.

### Histological analysis

Freshly collected tissues were fixed in 10% neutral buffered formalin, paraffin-embedded, and sectioned for histological staining. Liver sections were additionally stained with Oil Red O to visualize lipid droplets. All stained slides (hematoxylin and eosin (H&E) for general morphology; Oil Red O for hepatic lipids) were examined under a light microscope (Olympus, Tokyo, Japan). In quantitative analysis, we used ImageJ to measure the average area of epididymal adipocytes and the percentage of lipid droplet area in liver tissue stained with Oil Red O.

### Determination of mRNA expression levels

Hepatic and colonic mRNA levels of target genes (*PPARα*, *PGC1α*, *ACOX*, *HSL*, *CPT1*, *PGC1β*, *ACC*, *SREBP-1C*, *AP2*, *CD11*, *Sgk3*, *G6pc3*, *Mdm*, *Smad3*, *Pck1*, *Cdk2*, *Pbl2*, *Plk2*, *Ppkab1*, *Occludin, Claudin-1, Reg3b, Reg3g, IFN-γ*, *MCP-1*, *IL-1β, TNF-α*) were quantified *via* RT-qPCR. Total RNA was isolated using TaKaRa reagents (Japan), followed by concentration measurement and reverse transcription (Prime Script RT Kit with gDNA Eraser, TaKaRa). Amplification was performed on a Light Cycler 480 system (Roche, USA) with SYBR Green Master Mix (Takara). Gene expression normalization utilized the 2^−ΔΔCt^ method, with *β-Actin* and *RPL-19* as internal controls. Primer sequences (Supplementary Table S2) were designed and synthesized by Generay Biotech (China).

### Western blot analysis

Liver proteins were lysed in RIPA buffer. Protein quantification employed a BCA assay (Beyotime, China). After SDS-PAGE separation, proteins were transferred to PVDF membranes, blocked with 5% skim milk/TBST (1 h), and incubated overnight (4°C) with antibodies: PI3K (T40115F, Abmart, China), P-AKT (66444-1-Ig, Proteintech, China), AKT (10176-2-AP, Proteintech), AC-FoxO1 (AF2305, Affinity Biosciences, China), P-FoxO1 (9461T, Cell Signaling, USA), FoxO1 (2880 T, Cell Signaling), SREBP-1C (ab313881, Abcam, USA). After TBST washes, membranes were incubated with HRP-conjugated secondary antibodies (ABclonal, China). Protein bands were visualized *via* chemiluminescence (Biosharp, China) and analyzed using ImageJ.

### RNA-seq

Liver RNA was extracted, quality-checked (concentration/purity), and subjected to poly(A) selection (Oligo dT beads). mRNA fragmentation, cDNA synthesis, adapter ligation, and PCR amplification were performed to construct sequencing libraries, which were sequenced on an Illumina platform. Raw reads were aligned to reference genomes, and gene expression levels were calculated based on read counts. DEGs were identified and functionally annotated *via* GO and KEGG enrichment analyses.

### Sequencing of 16S rRNA gene of microorganisms

Cecal DNA was extracted and amplified *via* PCR. PCR products were quantified (QuantiFluor™-ST, Promega), pooled equimolarly, and sequenced on an Illumina platform (paired-end reads). Raw data were demultiplexed, quality-filtered, and denoised (DADA2 pipeline) to generate amplicon sequence variants (ASVs). Taxonomic classification of ASVs was performed using SILVA database.

### Statistical analysis

Data are presented as mean ± SEM. One-way ANOVA with Tukey’s post hoc test (GraphPad Prism 8.0, USA) was used for group comparisons. All figures were created using GraphPad Prism version 8.0 and assembled using Adobe Illustrator version 2020.

## Supplementary information

Supplementary materials

## Data Availability

The sequences generated in this study are stored in the National Center for Biotechnology Information (NCBI) and the project numbers are PRJNA1234563 and PRJNA1235473.
