# Supplementary material for: Integration of transcriptomics and gut microbiomics reveals walnut septum polyphenols alleviate HFD-induced lipid disorders
Source: NPJ Sci Food. 2026 Mar 26;10:175. doi: 10.1038/s41538-026-00801-y (PMC13230880; doi:10.1038/s41538-026-00801-y)
Supplement: Supplementary file 1 — Supplementary materials [file 41538_2026_801_MOESM1_ESM.docx]

**Integration of transcriptomics and gut microbiomics reveals walnut septum polyphenols alleviate HFD-induced lipid disorders**

Yue-Xiu Pan^1,2,#^, Lei Peng^1,3,#^, Xia Hu^1,3,4^, Jin-lian Chen^1,3,4^, Min Su^1,3,4^, Jing-jing Dai^5^, Jun Sheng^1,3,4^, Zi-Shan Hong^6,*^, Jing Xie^1,3,4,*^ and Yang Tian^1,3,4,*^

^1^ *College of Food Science and Technology, Yunnan Agricultural University, Kunming 650201, China*

^2^ *College of Pharmacy*, *Shan Dong Xian Dai University*, *Jinan 250104, China*

^3^ *Engineering Research Center of Development and Utilization of Food and Drug Homologous Resources, Ministry of Education, Yunnan Agricultural University, Kunming 650201, China*

^4^ *Yunnan Provincial Key Laboratory of Precision Nutrition and Personalized Food Manufacturing, Yunnan Agricultural University, Kunming 650201, China*

^5^ *School of Tea and Coffee,* *Puer University, Puer 665000, China*

^6^ *School of Food Science and Engineering, South China University of Technology, Guangzhou 510640, China*

**Author contributions**

Yuexiu Pan and Lei Peng contributed equally to this study.

*** Correspondence**

Prof. Yang Tian

Tel: +86-15925200355; Fax: +86-0871-65227843; E-mail: [tianyang1208@163.com](mailto:tianyang1208@163.com).

Assoc. Prof. Jing Xie

Tel: +86-18288759015; Fax: +86-0871-65227843; E-mail: [jingxie0624@163.com](mailto:jingxie0624@163.com).

Dr. Zishan Hong

E-mail: 15287190113@163.com

**Supplementary Table Captions**

**Table S1** The specific composition of the feed

**Table S2** Mice primer sequences in RT-qPCR assays

**Supplementary Table S1**

**Table S1** The specific composition of the feed

| **Ingredients** | **TP23520(g/kg)** | **TP23522(g/kg)** |
| --- | --- | --- |
| Casein | 258 | 190 |
| L-cystine | 4 | 3 |
| Corn starch | 0 | 480 |
| Dextrin | 162 | 118 |
| Sucrose | 89 | 65 |
| Soybean oil | 32 | 24 |
| Lard | 317 | 19 |
| Cellulose | 65 | 47 |
| Mineral mix | 58 | 43 |
| Vitamin mix | 13 | 9 |
| Choline bitartrate | 3 | 2 |
| Total | 1000 | 1000 |
| Energy calculated |  |  |
| Energy density,kcal/g | 5.2 | 3.9 |
| Energy distribution |  |  |
| Protein,% | 20% | 20% |
| Carbohydrate,% | 20% | 70% |
| Fat,% | 60% | 10% |
| Total | 100% | 100% |

**Supplementary Table S2**

**Table S2** Mice primer sequences in RT-qPCR assays

| **Genes** | **Forward** | **Reverse** |
| --- | --- | --- |
| *Sgk3* | AGAAAACAGCCCTATGACAACAC | AGCAACATCTCGGCAGTAAAA |
| *Rbl2* | TTAGCATGTGCCTTATATGTGGC | CTCCGAACAGCGAAGGATTC |
| *Pck1* | TGACAGACTCGCCCTATGTG | CCCAGTTGTTGACCAAAGGC |
| *Cdk2* | CCTGCTTATCAATGCAGAGGG | GTGCTGGGTACACACTAGGTG |
| *Smad3* | TCTCCCCGAATCCGATGTCC | GCTGGTTCAGCTCGTAGTAGG |
| *G6pc3* | TGCATGAATCCGGGTACTCCA | GGCCGTCATTACAGGCCAAA |
| *Mdm2* | TGTCTGTGTCTACCGAGGGTG | TCCAACGGACTTTAACAACTTCA |
| *Prkab1* | GACGCCGACATCTTCCACTC | GGGCTTTATCATTCGCTTCCAG |
| *Plk2* | AGCCAGAAGTCCGATACTACC | TCCCTAGCTTGAGATCCCTGT |
| *Pparα* | CAACGGCGTCGAAGACAAA | TGACGGTCTCCACGGACAT |
| *Pgc1α* | CCTGGCAAAGCATTTGTATG | TGGTCCAGAGAGTGCTTGTG |
| *Acox1* | CTATGGGATCAGCCAGAAAGG | AGTCAAAGGCATCCACCAAAG |
| *HSL* | TTCTCCAAAGCACCTAGCCAA | TGTGGAAAACTAAGGGCTTGTTG |
| *Cpt-1* | GCTGGAGGTGGCTTTGGT | GCTTGGCGGATGTGGTTC |
| *PGC1β* | CCTTCCACCTGAGCTATGGA | TATTGGAAGGGCCTTGTCTG |
| *Acc* | TGTTGAGACGCTGGTTTGTAGAA | GGTCCTTATTATTGTCCCAGACGTA |
| *SREBP1-C* | CTCAGGTACTGTTGGAAACC | AGACAGGGAGTTCTCAGATG |
| *AP2* | TCACCTGGAAGACAGCTCCT | AATCCCCATTTACGCTGATG |
| *IL-1β* | TCCATGAGCTTTGTACAAGGA | AGCCCATACTTTAGGAAGACA |
| *IL-10* | AAGGACCAGCTGGACAACAT | TCTCACCCAGGGAATTCAAA |
| *TNF-α* | AGACCCTCACACTCAGATCA | TCTTTGAGATCCATGCCGTTG |
| *INF-γ* | ATCTGGAGGAACTGGCAAAA | TTCAAGACTTCAAAGAGTCTGAG |
| *MCP-1* | TTAAAAACCTGGATCGGAACCAA | GCATTAGCTTCAGATTTACGGGT |
| *Claudin-1* | TGCCCCAGTGGAAGATTTACT | CTTTGCGAAACGCAGGACAT |
| *Reg3b* | CCCAGGCTTATGGCTCCTAC | ATGGAGCCCAATCCAAGTGT |
| *Reg3g* | TTCCTGTCCTCCATGATCAAA | CATCCACCTCTGTTGGGTTC |
| *Occludin/* | CAGCCTCGGTACAGCAGCAAT | ATAGTGGTCAGGGTCCGTCCTC |
| *β-actin* | ACGGCCAGGTCATCACTATTG | TGGAAAAGAGCCTCAGGGC |
| *RPL-19* | GCTTGCCTCTAGTGTCCTCC | TTGGCGATTTCATTGGTCTCA |
| *Muc2* | ACGTGTCATATTTGCACCTCT | TCAACATTGAGAGTGCCAACT |

**Supplementary Figure caption**

**Figure S1** Organ-to-body weight ratios in mice. (A) Heart, (B) lung and (C) kidney (n = 12). Significant difference is indicated by ## (*p* < 0.01). Statistical analyses were performed using one-way ANOVA followed by Tukey's multiple comparison test.

**Figure S2** The mRNA expression levels of *IFN-γ*, *MCP-1*, *IL-1β*, *TNF-α*, *Occludin*, *Claudin-1*, *Reg3b* and *Reg3g* in liver tissues were determined by RT-qPCR (n = 6). Data are mean ± SEM. #*p* < 0.05, ##*p* < 0.01, ###*p* < 0.001 and ####*p* < 0.0001 for comparisons between NCD and HFD groups. **p* < 0.05, ***p* < 0.01, ****p* < 0.001, *****p* < 0.0001, for comparisons between HFD and HFD + HWSP groups. Statistical analyses were performed using one-way ANOVA followed by Tukey's multiple comparison test.

**Figure S3** The number of differential genes (DGEs) between the NCD vs HFD, NCD vs HFD + HWSP and HFD vs HFD + HWSP groups (A) and the volcano plots of differential gene expression between the NCD vs HFD (B) and HFD vs HFD + HWSP (C) groups were determined by transcriptome analysis of liver tissues.

**Figure S4** WSP modulates high-fat diet (HFD)-induced changes in gut flora at the family level. (A) Structural composition of the community at the family level. (B) Comparison of the relative abundance of different microbial communities at the family level among the NCD, HFD, and HFD+HWSP groups. **p* < 0.05, ***p* < 0.01, ****p* < 0.001, *****p* < 0.0001. Statistical analyses were performed using one-way ANOVA followed by Tukey's multiple comparison test.

**Figure S5** WSP modulates high-fat diet (HFD)-induced changes in gut flora at the genus-level. (A) Structural composition of the community at the genus level. (B) Comparison of the relative abundance of different microbial communities at the genus level among the three groups of NCD, HFD, and HFD+HWSP. **p* < 0.05, ***p* < 0.01, ****p* < 0.001, *****p* < 0.0001. Statistical analyses were performed using one-way ANOVA followed by Tukey's multiple comparison test.

**Figure S6** PICRUSt2 functional prediction heat map and representative module plots of the level 2 pathways.

**Supplementary Figure S1**


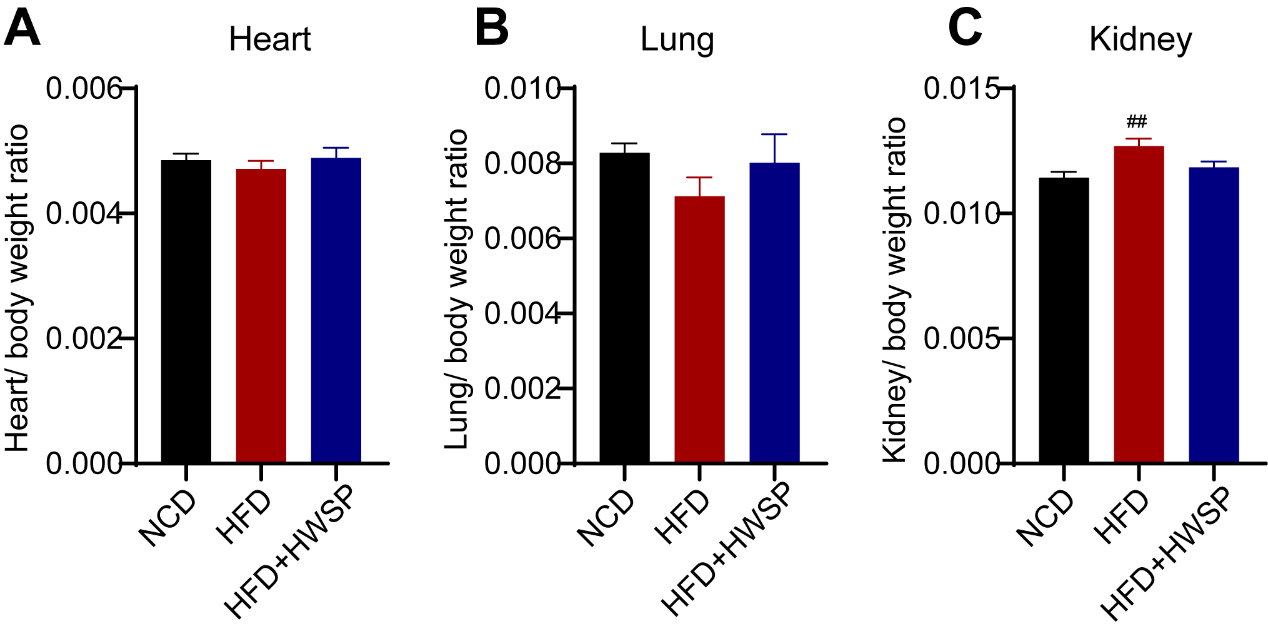


**Figure S1** Organ-to-body weight ratios in mice. (A) Heart, (B) lung and (C) kidney (n = 12). Significant difference is indicated by ## (*p* < 0.01). Statistical analyses were performed using one-way ANOVA followed by Tukey's multiple comparison test.

**Supplementary Figure S2**


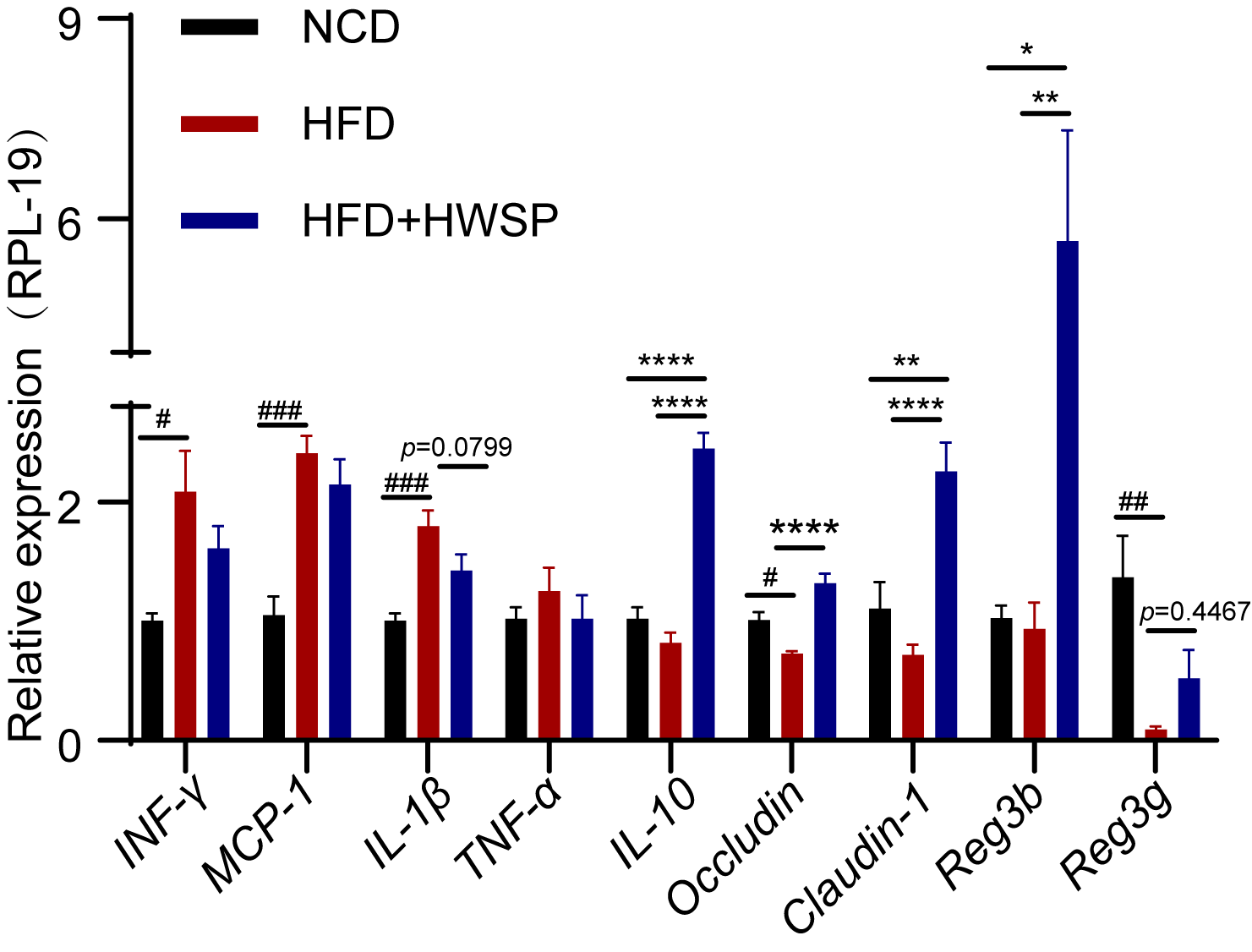


**Figure S2** The mRNA expression levels of *IFN-γ*, *MCP-1*, *IL-1β*, *TNF-α*, *Occludin*, *Claudin-1*, *Reg3b* and *Reg3g* in liver tissues were determined by RT-qPCR (n = 6). Data are mean ± SEM. #*p* < 0.05, ##*p* < 0.01, ###*p* < 0.001 and ####*p* < 0.0001 for comparisons between NCD and HFD groups. **p* < 0.05, ***p* < 0.01, ****p* < 0.001, *****p* < 0.0001, for comparisons between HFD and HFD + HWSP groups. Statistical analyses were performed using one-way ANOVA followed by Tukey's multiple comparison test.

**Supplementary Figure S3**


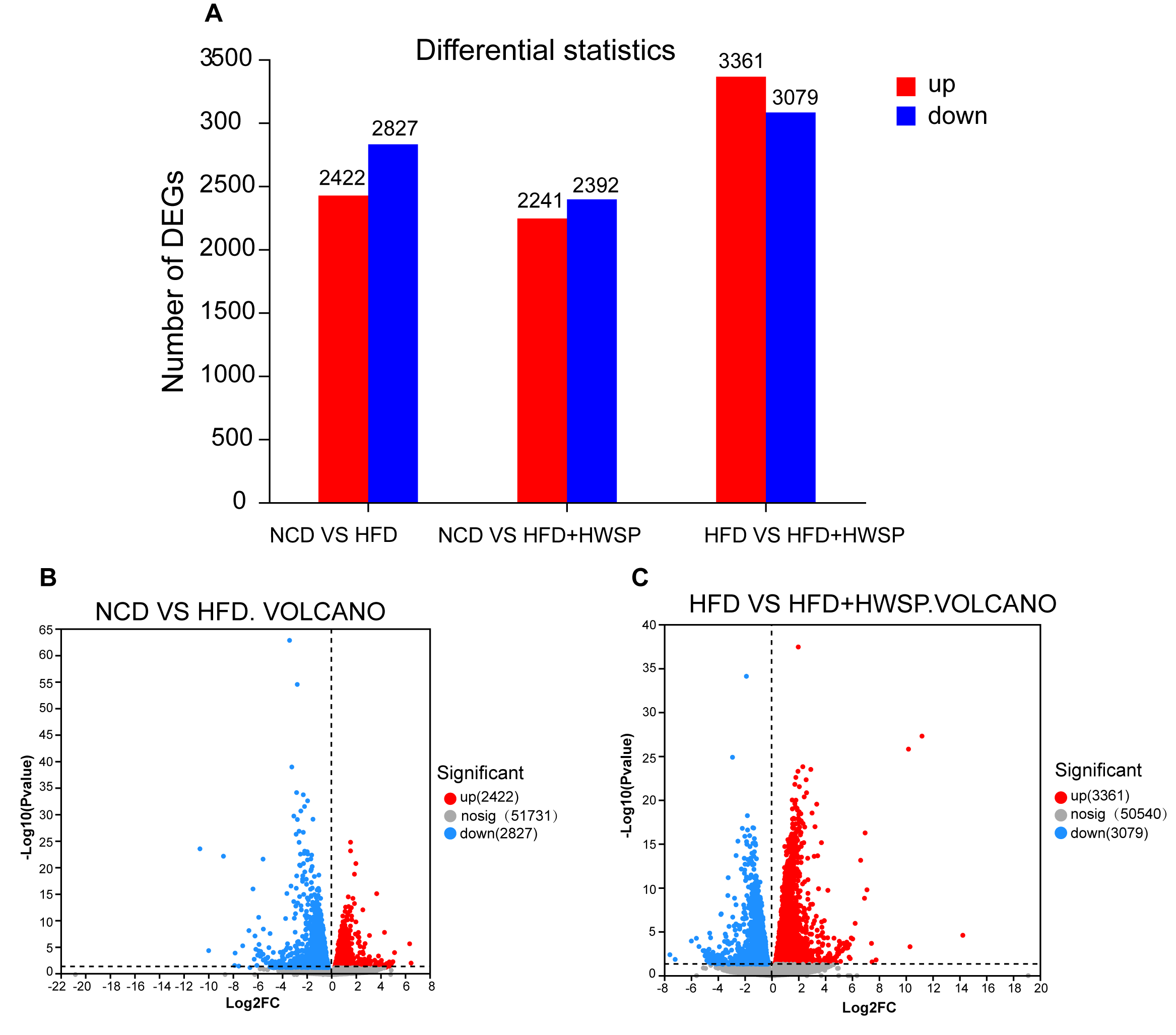


**Figure S3** The number of differential genes (DGEs) between the NCD vs HFD, NCD vs HFD + HWSP and HFD vs HFD + HWSP groups (A) and the volcano plots of differential gene expression between the NCD vs HFD (B) and HFD vs HFD + HWSP (C) groups were determined by transcriptome analysis of liver tissues.

**Supplementary Figure S4**

**
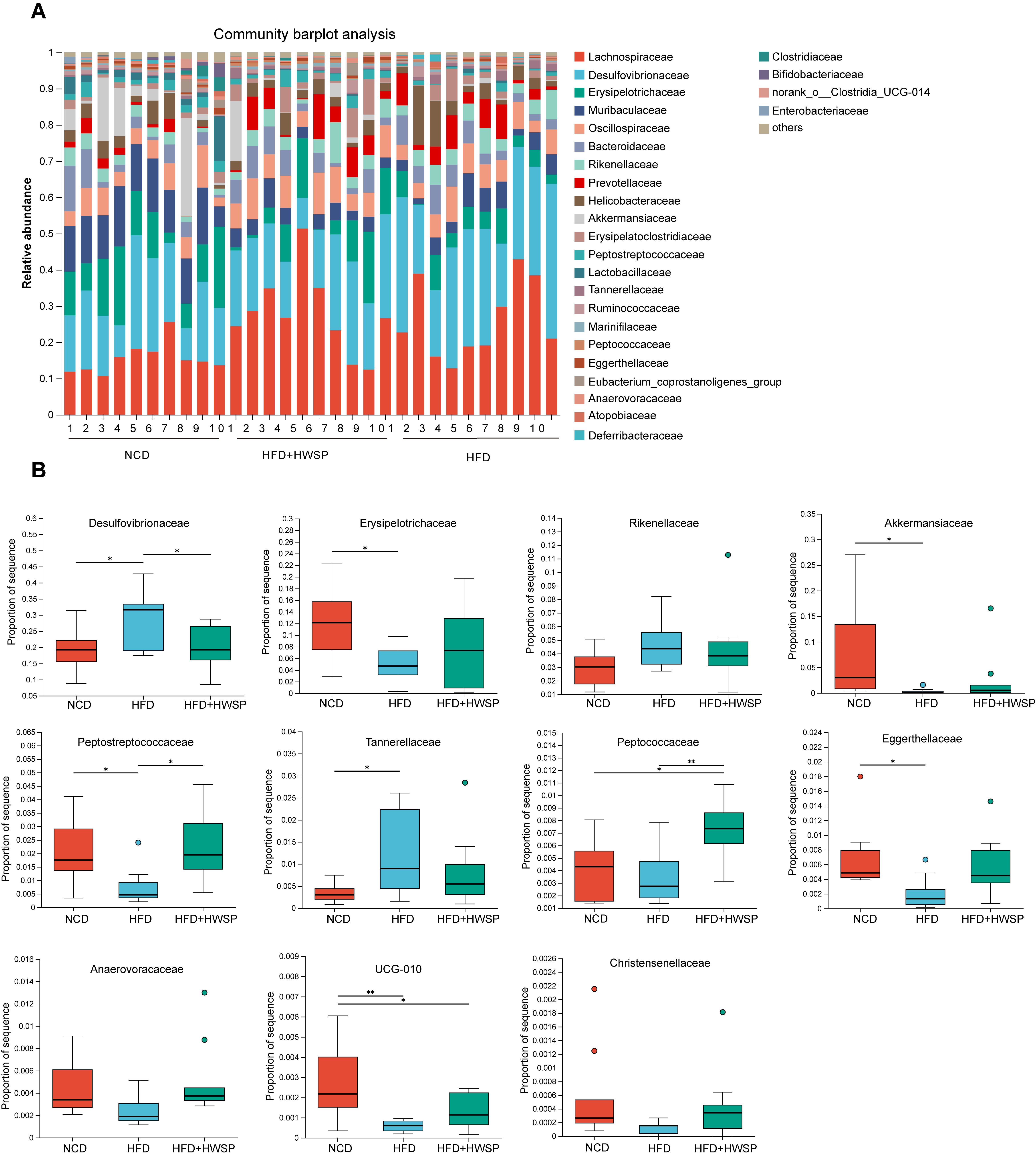
**

**Figure S4** WSP modulates high-fat diet (HFD)-induced changes in gut flora at the family level. (A) Structural composition of the community at the family level. (B) Comparison of the relative abundance of different microbial communities at the family level among the NCD, HFD, and HFD+HWSP groups. **p* < 0.05, ***p* < 0.01, ****p* < 0.001, *****p* < 0.0001. Statistical analyses were performed using one-way ANOVA followed by Tukey's multiple comparison test.

**Supplementary Figure S5**


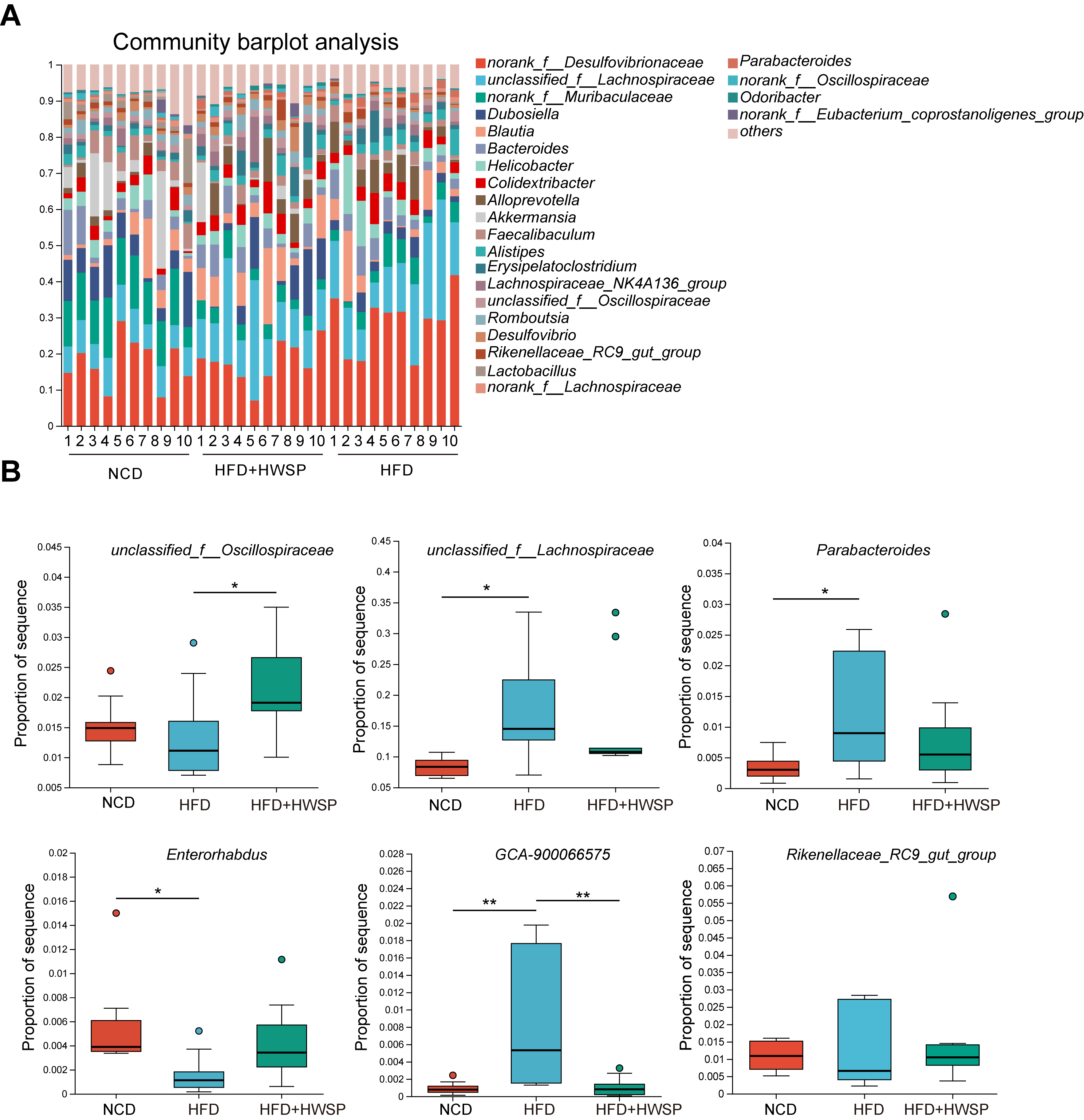


**Figure S5** WSP modulates high-fat diet (HFD)-induced changes in gut flora at the genus-level. (A) Structural composition of the community at the genus level. (B) Comparison of the relative abundance of different microbial communities at the genus level among the three groups of NCD, HFD, and HFD+HWSP. **p* < 0.05, ***p* < 0.01. Statistical analyses were performed using one-way ANOVA followed by Tukey's multiple comparison test.

**Supplementary Figure S6**


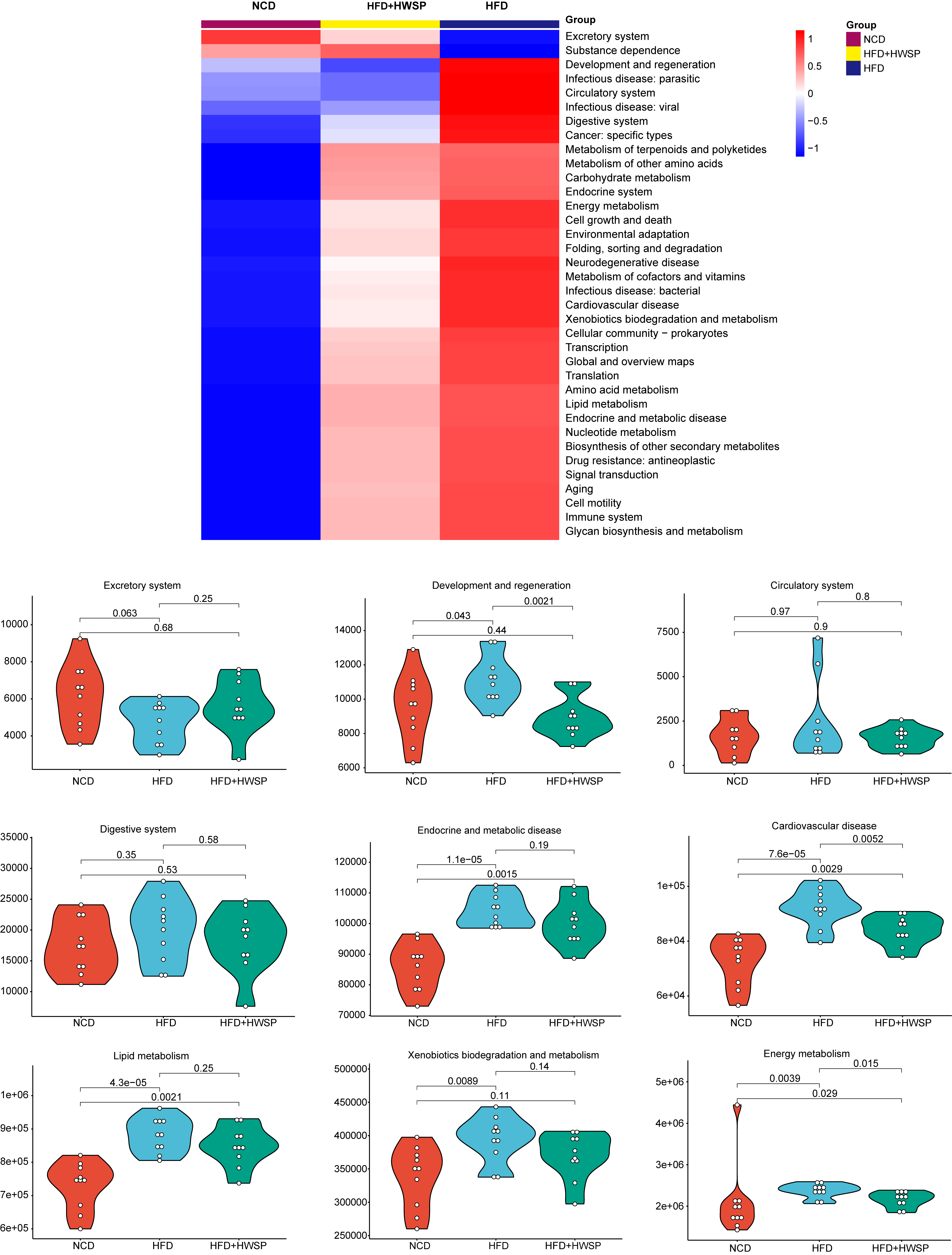


**Figure S6** PICRUSt2 functional prediction heat map and representative module plots of the level 2 pathways.
